# Hibiscus Chlorotic Ringspot Virus Coat Protein Is Essential for Cell-to-Cell and Long-Distance Movement but Not for Viral RNA Replication

**DOI:** 10.1371/journal.pone.0113347

**Published:** 2014-11-17

**Authors:** Shengniao Niu, Francisco M. Gil-Salas, Sunil Kumar Tewary, Ashwin Kuppusamy Samales, John Johnson, Kunchithapadam Swaminathan, Sek-Man Wong

**Affiliations:** 1 Department of Biological Sciences, National University of Singapore, Singapore, Singapore; 2 Key Laboratory of Tropical Crop Biotechnology, Ministry of Agriculture, Institute of Tropical Bioscience and Biotechnology, Chinese Academy of Tropical Agricultural Science, Hainan, China; 3 Instituto Andaluz de Investigación y Formación Agraria, Pesquera, Alimentaria y de la Producción Ecológica, Almería, Spain; 4 Department of Molecular Biology, The Scripps Research Institute, California, United States of America; 5 Temasek Life Sciences Laboratory, Singapore, Singapore; 6 National University of Singapore Suzhou Research Institute, Suzhou Industrial Park, Jiangsu, China; USDA-ARS, United States of America

## Abstract

*Hibiscus chlorotic ringspot virus* (HCRSV) is a member of the genus *Carmovirus* in the family *Tombusviridae*. In order to study its coat protein (CP) functions on virus replication and movement in kenaf (*Hibiscus cannabinus* L.), two HCRSV mutants, designated as p2590 (A to G) in which the first start codon ATG was replaced with GTG and p2776 (C to G) in which proline 63 was replaced with alanine, were constructed. *In vitro* transcripts of p2590 (A to G) were able to replicate to a similar level as wild type without CP expression in kenaf protoplasts. However, its cell-to-cell movement was not detected in the inoculated kenaf cotyledons. Structurally the proline 63 in subunit C acts as a kink for β-annulus formation during virion assembly. Progeny of transcripts derived from p2776 (C to G) was able to move from cell-to-cell in inoculated cotyledons but its long-distance movement was not detected. Virions were not observed in partially purified mutant virus samples isolated from 2776 (C to G) inoculated cotyledons. Removal of the N-terminal 77 amino acids of HCRSV CP by trypsin digestion of purified wild type HCRSV virions resulted in only T = 1 empty virus-like particles. Taken together, HCRSV CP is dispensable for viral RNA replication but essential for cell-to-cell movement, and virion is required for the virus systemic movement. The proline 63 is crucial for HCRSV virion assembly in kenaf plants and the N-terminal 77 amino acids including the β-annulus domain is required in T = 3 assembly *in vitro*.

## Introduction

*Hibiscus chlorotic ringspot virus* (HCRSV) belongs to the genus *Carmovirus* in the family *Tombusviridae* [1], [2] and is distributed worldwide in hibiscus [3], [4]. It can be mechanically inoculated onto host plants and it is not known if it can be transmitted through seeds [5]. Infected plants show symptoms of generalized mottle to chlorotic ringspot and vein banding patterns [6]. HCRSV is icosahedral in shape with a diameter of approximately 30 nm [7] and contains a single-stranded positive-sense RNA genome of 3911 nt and produces two subgenomic RNAs (sgRNAs) during replication [8], [9].

The structures of several carmoviruses, such as *Turnip crinkle virus* (TCV) [10], *Carnation mottle virus* (CarMV) [11], *Cowpea mottle virus* (CPMoV) [12] and *Melon necrotic spot virus* (MNSV) [13], have been reported. For HCRSV, we have earlier reported a low resolution structure, determined by cryo-electron microscopy image reconstruction [7]. In an earlier crystallization attempt, HCRSV crystals diffracted to 4.5 Å resolution [14]. Later, we solved the structure at 3.2 Å resolution [15]. The coordinates are deposited at the Viper database (viperdb.scripps.edu/info_page.php?VDB = hcrsv). The structure shows that the virion is formed by 180 copies of identical coat protein (CP) subunit, arranged with a T = 3 quasi-symmetry composed of A, B and C subunit types, similar to other *Tombusviridae* members [16]. Each CP subunit has three domains: the RNA-binding (R), the shell forming (S) and the protruding (P) domain [15]. The structure formed by the N-terminal projection in the R domain of HCRSV CP around the icosahedral three-fold symmetry is referred to as β-annulus, such feature is also present in other T = 3 icosahedral viruses including *Tombusvirus*, *Bromovirus* and *Sobemovirus* [17]. Deletion of the N-terminal 22 amino acids of *Sesbania mosaic virus* (SeMV) CP *in vitro* does not affect T = 3 capsid assembly, but further deletion to residue 36, containing an arginine-rich motif (ARM), results in two distinct capsids, T = 1 and pseudo T = 2. Only the T = 1 particles were observed upon deletion of N-terminal 65 amino acids. Another study on the role of the β-annulus of *Cucumber necrosis virus* (CNV) showed that deletion mutant of the β-annulus is capable of producing virus particles in plants [18]. Similar result was observed in *Turnip yellow mosaic virus* (TYMV), in which the native β-annulus is not essential for infectious particle assembly, although its deletion will reduce the yield of viral particles [19].

In the HCRSV structure, a β-annulus between isoleucine 53 and proline 63 in C subunits was clearly shown (viperdb.scripps.edu/info_page.php?VDB = hcrsv). The conserved proline 63 is a kink for the β-annulus formation. In SeMV, replacement of its conserved proline 53 with alanine can still form T = 3 virus-like particles (VLPs) *in vitro* [20] and the bending and β-annulus structure are not affected [21]. Similar replacement of the proline 85 with glycine in CNV also results in T = 3 and T = 1 particles *in vivo* [22]. In bacteriophage MS2, replacement of its conserved proline 78 with asparagine allows the expressed CP in *E. coli* to form T = 3 VLP [23]. However, the same mutation in the full-length cDNA clone of MS2 is unable to produce infectious particles in *E. coli* [24].

HCRSV CP, in addition to its structural role, also plays non-structural roles in gene silencing suppression [25] and interaction with host proteins [26]. To study functions of the CP in cell-to-cell and long-distance movement, and function of the proline 63 in virion assembly, two HCRSV mutants were constructed. Our results showed that HCRSV CP is not required for viral RNA replication but essential for cell-to-cell movement. The proline 63 is essential for virion assembly which in turn determines systemic movement of the virus. Removal of the N-terminal 77 amino acids including the β-annulus domain by limited trypsin digestion of swollen HCRSV virions resulted in T = 1 empty VLPs.

## Materials and Methods

### Construction of HCRSV Mutants and *in Vitro* Transcription

Mutant p2590 (A to G), in which HCRSV CP start codon ATG was changed to GTG, was first amplified using primer pair HC2590-F/HC2590-R (all the primers used in this study are listed in Table 1) with the wild type (wt) construct p223 [8] as template. The PCR product was then cloned into the pGEM-T Easy Vector (Promega). After sequencing, a subclone with correct insert was digested with the *Hpa*I and *Bsr*GI restriction enzymes, followed by insertion into p223. Mutants p2776 (C to G), p2590 (A to G)-ACG and p2590 (A to G)-ACA were made by site directed mutagenesis with primer pairs HC2776-F/HC2776-R, HC2590-ACG-F/HC2590-ACG-R and HC2590-ACA-F/HC2590-ACA-R, respectively, using p223 or p2590 (A to G) as template. To perform *in vitro* transcription, these mutants and p223 digested with *Sma*I were transcribed with MEGAscript T7 kit (Life Technologies, Ambion). To obtain cDNAs of HCRSV subgenomic RNA 2 with or without mutation, primers HC-F12 and HC-R3 were used to perform PCR using p2590 (A to G), p2590 (A to G)-ACG, p2590 (A to G)-ACA, p2776 (C to G) and p223 as templates, respectively. For deletion of the region between the first and second ATG of CP gene, primer pairs HC-F12/HC-R12 M and HC-F16/HC-R3 were used to perform PCR, respectively, using p2590 (A to G) as template, followed by overlap PCR using primers HC-F12 and HC-R3. These PCR products were used for *in vitro* transcription, followed by *in vitro* translation in wheat germ extract (Promega). The translation products were labeled with biotinylated lysine (Transcend tRNA, Promega).

**Table 1:** Oligonucleotides used in this study.

Primer	Sequence (5′-3′)^a^	Structureand/or position^b^
HC2590-F	GTTAACACTGGAAAGAAACCAACCACAGTG	2562 to 2591
HC2590-R	TGTACACTATTTTGGGAGTCCGATCG	3765 to 3740
HC2776-F	CACTTAAGGTTACAGCTGCGGTGGCCGCCTCGATG	2758 to 2792
HC2776-R	CATCGAGGCGGCCACCGCAGCTGTAACCTTAAGTG	2792 to 2758
HC-F8	TGGGATGGAGGTGAAGCAGA	3486 to 3505
HC-R3	AAGGGCTGCCTCACAACTATGG	3911 to 3892
HC2590-ACG-F	GGTGGCCGCCACGATGCGGACTCGAAACCCTGGAGCAAAC	2777 to 2816
HC2590-ACG-R	CCGCATCGTGGCGGCCACCGGAGCTGTAACCTTAAGTGTTG	2795 to 2755
HC2590-ACA-F	GGTGGCCGCCACAATGCGGACTCGAAACCCTGGAGCAAAC	2777 to 2816
HC2590-ACA-R	CCGCATTGTGGCGGCCACCGGAGCTGTAACCTTAAGTGTTG	2795 to 2755
HC-F12	taatacgactcactataGGGAAAATTGCTTTATCATAACC	T7, 2438 to 2460
HC-R12 M	GCTCCAGGGTTTCGAGTCCGCATTGTGGTTGGTTACTTTCCA	2814 to2791, 2589 to 2570
HC-F16	ATGCGGACTCGAAACCCTGGAGC	2791 to 2814
HC-F5	ggatccATGCTCTCACATGCTTTCTC	*Bam*HI, 41 to 60
HC-R5	ctgcagTCACGGGCGAGTACCCCTGA	*Pst*I, 670 to 650

a Upper case, upper case with underlined and lower case refer to HCRSV cDNA nucleotides, mutated HCRSV cDNA nucleotides and added sequences (T7 promoter or restriction enzyme sites), respectively.

b Numbers correspond to HCRSV nucleotide positions.

### Transfection and Inoculation of Kenaf Protoplasts and Cotyledons

Kenaf seeds (cultivar Everglades 41) were obtained from Mississippi State University, U.S.A. Protoplasts (9×10^5^) isolated from kenaf seedlings as described [27] were transfected with 10 µg of transcripts each derived from *Sma*I digested p2590 (A to G), p2776 (C to G) and p223 in triplicates, respectively. After transfection, all protoplasts from the triplicates were pooled and incubated in MS medium and harvested at 24 and 48 hours post transfection (hpt) for total RNA extraction. Protoplasts harvested at 72 hpt were used for total protein extraction. Kenaf cotyledons from 2-week-old seedlings were inoculated with the same transcripts (0.5 µg for each cotyledon). Two cotyledons were harvested and pooled for total RNA extraction at 1, 2 and 3 days post inoculation (dpi). Upper leaves at 25 dpi were also harvested for total RNA extraction.

### Northern Blot and RT-PCR

Total RNA was extracted as described [28] and separated in 1.5% TBE agarose gel (2.5 µg for protoplasts and 5 µg for leaves) and transferred onto positively charged nylon membrane using alkaline transfer solution (3 M NaCl, 0.01 N NaOH). Hybridization was carried out according to the instructions (Roche) and signal was developed using substrates NBT/BCIP. A 425 bp DIG-labeled PCR products amplified by using primer pair HC-F8/HC-R3 was used as probe. For RT-PCR, primer HC-R3 was used for cDNA synthesis and primer pair HC-F8/HC-R3 were used for PCR amplification.

### Total Protein Extraction and Western Blot

Plant tissues were homogenized in liquid nitrogen and protein extraction buffer [220 mM Tris–HCl, pH 7.4, 250 mM sucrose, 50 mM KCl, 1 mM MgCl_2_, 2 mM phenylmethylsulfonyl fluoride (PMSF)] was added at 1∶5 ratio (w/v) once the homogenized tissue powder was thawed. After removing plant debris by centrifugation at 4°C, proteins in supernatant were denatured by heating in a boiling water bath for 5 min in 2×SDS gel-loading buffer. SDS-PAGE was performed and transferred to nitrocellulose membrane. Polyclonal HCRSV rabbit antiserum and alkaline phosphatase conjugated goat anti-rabbit IgG (Sigma) secondary antibody were used. Immuno-detection of proteins was carried out by the NBT/BCIP detection system (Roche).

### Purification of Virions from Inoculated Kenaf Cotyledons

Virions were purified as described [29] with slight modifications. Twenty four kenaf cotyledons (approximate 3 g) inoculated with each transcript at 5 dpi were collected and homogenized in liquid nitrogen to fine powder. After the powder was thawed, 40 ml of 0.2 M NaOAc (pH 5.2) was added to the powder, followed by passage through 4 layers of cheese clothes. The supernatant (after centrifugation at 7,700×g, 4°C) was layered onto 20% sucrose cushion, followed by centrifugation at 146,000×g for 2 h. Pellets were resuspended in 500 µl of 10 mM Tris (pH 7.3), followed by 3 rounds of centrifugation (12,000×g for 10 min each) to remove remaining impurities. The supernatants were used for western blot and negative staining with 2% uranyl acetate, followed by observation of virus particles using a transmission electron microscopy (TEM, model JEOL JEM 2010F HRTEM). To eliminate the possibility of high pH to virion stability, virions were also purified from same amount of inoculated cotyledons using lower pH in sucrose cushion [20% sucrose in 0.2 M NaOAc (pH 5.2)] and virus resuspension buffer (0.05 M NaOAc pH 5.4, 50 mM NaCl, 20 mM CaCl_2_, 5 mM EDTA) [15].

### Trypsin Digestion and Reassembly of HCRSV particles

HCRSV virions (60 µg), purified from infected kenaf plants as described [15], were incubated in 80 µl of virion swelling buffer (200 mM Tris-Cl, pH 7.5, 30 mM EDTA) for 40 min at room temperature [30] to obtain swollen virions, followed by trypsin digestion as described with slight modifications [31], [32]. Firstly, 800 µl of 25 mM NH_4_HCO_3_ containing 2 µg of sequencing grade modified trypsin (Promega) were added into the swollen virions and incubated at room temperature. Twenty µl of reaction mix was taken at each time point, followed by adding equal volume of protein loading dye and immediately boiled for 5 min for 15% SDS-PAGE. To avoid over-digestion of CP, limited trypsin digestion was carried out. The swollen virions were incubated in 800 µl of 25 mM NH_4_HCO_3_ containing 2 µg trypsin at room temperature for 5 min. The reaction mixture was then put on ice for 10 min, followed by incubation overnight at 4°C. To stop the reaction, 4 µl of aprotinin (Sigma) and 8 µl of 100 mM PMSF (Sigma) were added to the trypsin digested virions solution. The solution was then dialyzed as described [33]. After this, the assembled particles were pelleted by ultracentrifugation for 3 h at 125,000×g at 4°C. The pellet was resuspended in 100 µl of virus storage buffer [50 mM NaOAc pH 5.2, 8 mM Mg(OAc)_2_], followed by concentration using Microcon centrifugal filter (Millipore). The concentrated solution was used for negative staining with 2% uranyl acetate, followed by observation of virus particles under TEM.

### N-terminal Protein Sequencing

After limited trypsin digestion of the swollen HCRSV virioins, the concentrated preparation was resolved on 12% SDS-PAGE and transferred onto PVDF membrane, followed by staining with Coomassie blue staining solution. The protein band was cut out for N-terminal sequencing to determine the trypsin cleavage site.

## Results

### HCRSV CP does not Affect Viral RNA Replication in Protoplasts

**Figure 1:**
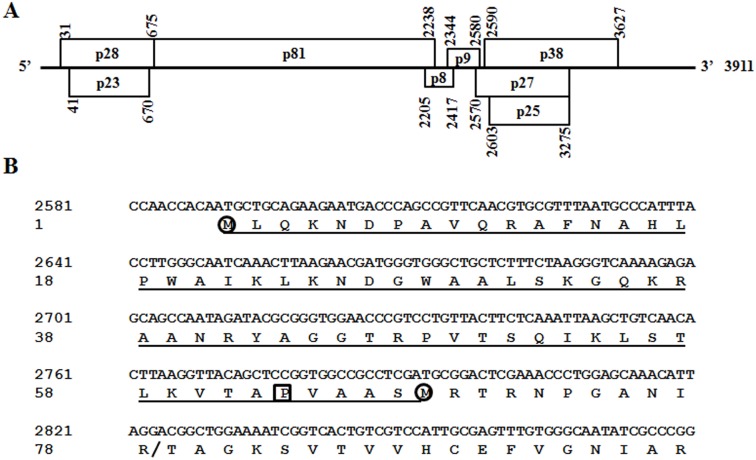
Schematic representations of (A) HCRSV genome and (B) partial HCRSV CP sequence showing mutation sites and deletion. Rectangles in (A) represent open reading frames. Underlined amino acids in (B) indicate the non-expressed portion as the CP start codon ATG was replaced with GTG in mutant p2590 (A to G) and the boxed proline (CCG) was substituted with alanine (GCG) to remove the kink of CP in mutant p2776 (C to G). The circled methionine residues are the translation initiation sites of CP in wt HCRSV (p223) and mutant p2590 (A to G). Symbol slash (/) represents the cleavage site when swollen HCRSV virions were digested with trypsin.

**Figure 2:**
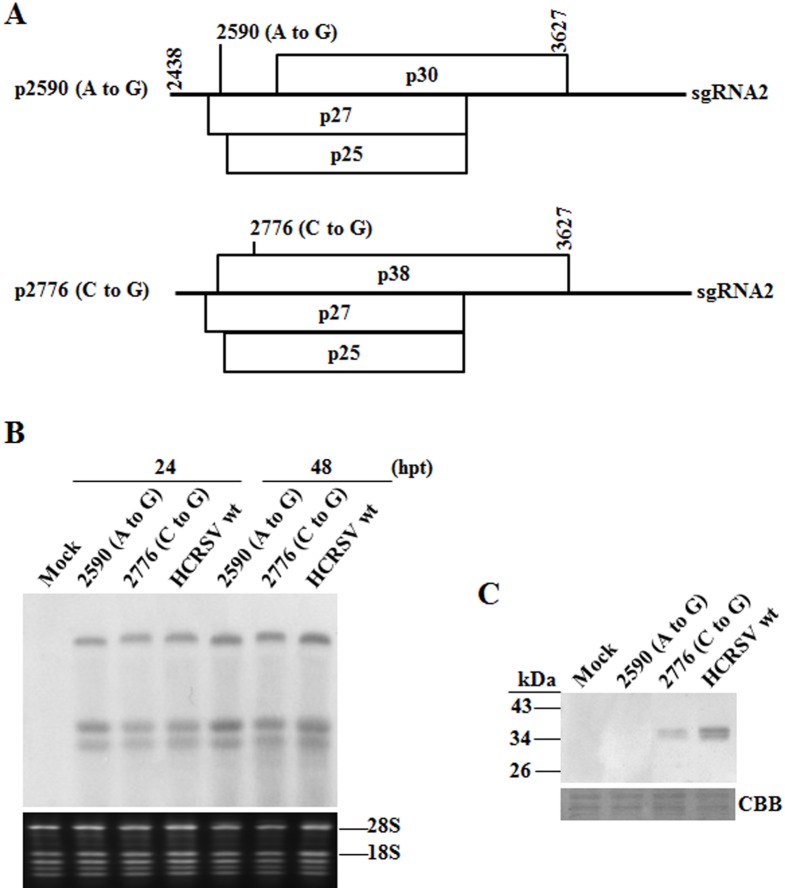
HCRSV RNA and CP accumulation in transfected kenaf protoplasts. (A) Schematic representation of mutant 2590 (A to G) and mutant 2776 (C to G). Only region covering sgRNA2 is shown to indicate the mutation sites. p30 is an ORF encoding a putative 30 kDa protein. (B) Northern blot analysis of viral RNA accumulation. *In vitro* transcripts (10 µg each) were transfected into 9×10^5^ protoplasts and harvested at different time points. Total RNA (2.5 µg each) extracted from protoplasts collected at 24 and 48 h post transfection (hpt), respectively, was used for viral RNA detection. DIG-labeled 425 bp HCRSV PCR product located in the 3′ region of the genome was used as the probe for hybridization. (C) Western blot analysis of HCRSV CP. Total protein was extracted from protoplasts collected at 72 hpt. CBB denotes Coomassie blue staining.

To test effects of HCRSV CP on virus replication, movement and assembly, two CP mutants were constructed using HCRSV cDNA full-length clone p223 as template (Fig. 1A) based on the deduced CP amino acids sequence (Fig. 1B), and designated as p2590 (A to G) and p2776 (C to G) (Fig. 2A). In mutant p2590 (A to G), the CP start codon was replaced with GTG so that the translation of CP will presumably start at the second ATG (methionine 68), resulting in a truncated 30 kDa protein without N-terminal 67 amino acids. In mutant p2776 (C to G), the CCG codon encoding proline 63 was changed to GCG (encoding an alanine) in order to study its effects on virion assembly. Transcripts derived from these two mutants and the wt clones were transfected into kenaf protoplasts. Northern blot showed that there was no significant difference in viral RNA accumulation among them at 24 and 48 hpt, respectively (Fig. 2B). To investigate the CP expression from these 2 mutants, total proteins were extracted from transfected protoplasts at 72 hpt and corresponding western blot showed that the truncated CP was not detected in mutant 2590 (A to G), whereas CP expression of mutant 2776 (C to G) was reduced, compared to that of HCRSV wt (Fig. 2C). These results indicate that HCRSV CP is dispensable for viral RNA replication in kenaf protoplasts.

### HCRSV CP is Essential for Cell-to-Cell Movement

**Figure 3:**
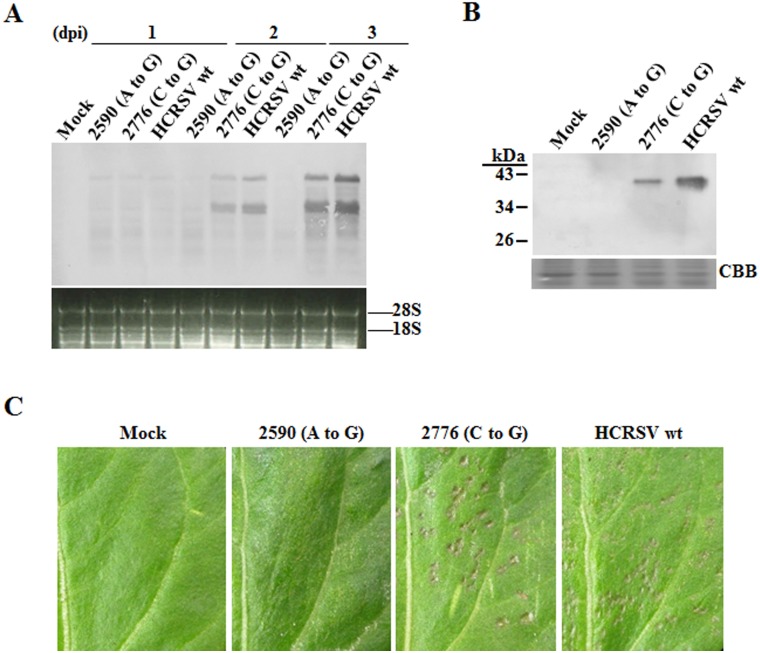
HCRSV viral RNA and CP accumulation in inoculated kenaf cotyledons. *In vitro* transcripts (0.5 µg for each cotyledon) were inoculated onto kenaf cotyledons and the inoculated cotyledons were collected at different time points. (A) Northern blot analysis of viral RNA. Total RNA (5 µg each) extracted from cotyledons at 1, 2 and 3 days post inoculation (dpi), respectively, was used for viral RNA detection. (B) Western blot analysis of viral CP. Total protein extracted from cotyledons at 4 dpi was used for HCRSV CP detection by western blot. (C) Observation for local lesions in inoculated kenaf cotyledons at 4 dpi.

To investigate effects of HCRSV CP on virus cell-to-cell movement, transcripts derived from the two HCRSV mutants were inoculated onto kenaf cotyledons. Viral RNA accumulation in mutant 2590 (A to G) inoculated cotyledons did not increase over time within 3 days, whereas the viral RNA accumulation increased over time in mutant 2776 (C to G) inoculated cotyledons, although its overall RNA level was slightly lower, as compared to the wt (Fig. 3A). Western blot showed that the CP accumulation in mutant 2776 (C to G) at 4 dpi was also lower than that in the wt, while an estimated 30 kDa truncated CP was undetected in mutant 2590 (A to G) (Fig. 3B). Two possible reasons for undetectable truncated CP were given and investigated in discussion section. At 4 dpi, local lesions were observed in wt and mutant 2776 (C to G) inoculated cotyledons but not in mutant 2590 (A to G) inoculated cotyledons (Fig. 3C). Therefore, the CP expression is required for HCRSV cell-to-cell movement, since efficient cell-to-cell movement of virus is essential for local lesion development [34], [35]. DNA sequencing of RT-PCR products from total RNA extracted from inoculated cotyledons at 4 dpi showed that the nucleotide sequence of the two mutants remained unchanged.

### Proline 63 in HCRSV CP is Essential for Virion Assembly

**Figure 4:**
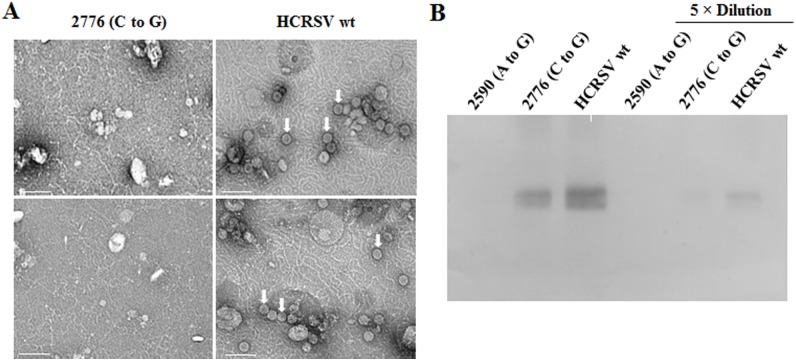
HCRSV virion assembly in inoculated kenaf cotyledons at 5 dpi. (A) Observation of virions under transmission electron microscope. Virus particles from inoculated cotyledons at 5 dpi were partially purified and negatively stained with 2% uranyl acetate. Partially purified virions were only obtained in HCRSV wt extract, regardless of using Tris pH 7.3 (top two panels) or sodium acetate pH 5.2 (bottom two panels) in sucrose cushion and resuspension buffer. Arrow heads point to virions. Each bar represents 100 nm. (B) Western blot analysis of HCRSV CP from the same extracts with or without dilution.

To determine whether virus assembly occurs in plants inoculated with mutant 2776 (C to G), kenaf cotyledons collected at 5 dpi were used for virion purification, followed by negative staining with 2% uranyl acetate. TEM results showed that no virion was observed in mutant 2590 (A to G) due to the lack of CP expression. Surprisingly, virions were not observed in mutant 2776 (C to G), as compared to that in the wt virus (Fig. 4A, top panels), although CP was detected in mutant 2776 (C to G) samples (Fig. 4B). Virions were still not observed in mutant 2776 (C to G) sample when low pH was used in sucrose cushion solution and resuspension buffer during virion purification (Fig. 4A, bottom panels). These results indicate that, without the proline 63 which presumably affects β-annulus formation, virions are not assembled in kenaf plants.

### Proline 63 Mutant of HCRSV is Unable to Infect Kenaf Plants Systemically

**Figure 5:**
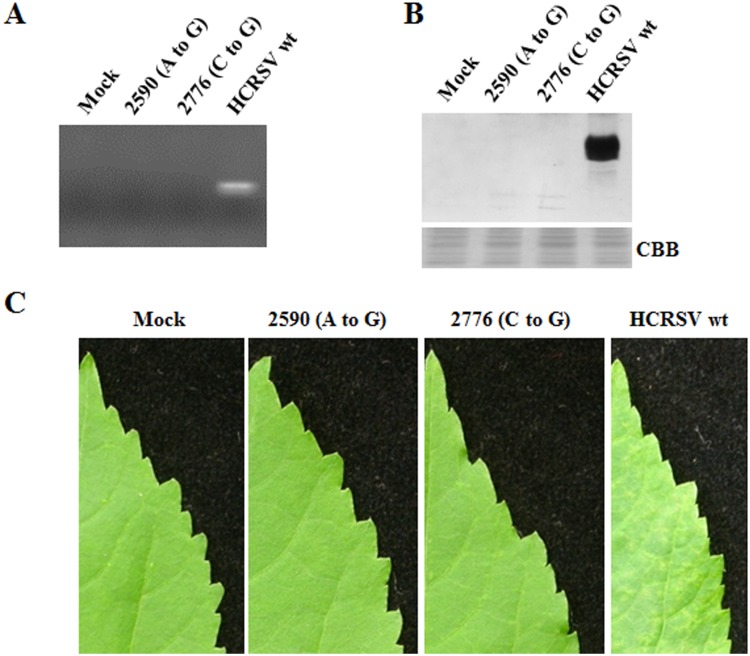
Detection of HCRSV RNA and its CP accumulation in upper leaves at 25 dpi. (A) Detection of viral RNA in upper leaves by RT-PCR using primer HC-R3, followed by PCR using primers HC-F8 and HC-R3 (Table 1). (B) Detection of HCRSV CP in upper leaves by western blot. (C) Symptoms of upper leaves of inoculated kenaf plants at 25 dpi.

Since mutant 2590 (A to G) was unable to move efficiently from cell to cell (Fig. 3), it is not surprising that its viral RNA was not detected in the upper leaves (Fig. 5A). We wonder whether long-distance movement occurs in mutant 2776 (C to G) inoculated plants without virion assembly, although CP was accumulated in its inoculated cotyledons (Fig. 3B). RT-PCR and western blot results showed that viral RNA and CP were not detected in the upper leaves of kenaf plants inoculated with transcripts of 2776 (C to G) at 25 dpi (Fig. 5A and B). Viral symptoms of chlorosis and mosaic were observed only in the upper leaves of plants inoculated with HCRSV wt transcripts (Fig. 5C). These results demonstrate that virion assembly is required for HCRSV long-distance movement.

### HCRSV CP without its N-terminal 77 Amino Acids Assembles into T = 1 Empty VLPs *in Vitro*

**Figure 6:**
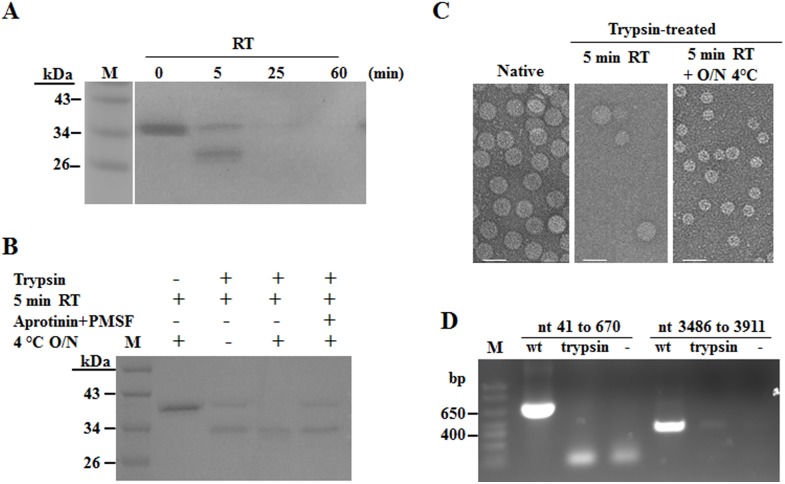
Re-assembly of HCRSV particles after trypsin digestion. (A) Time-course trypsin digestion of HCRSV virions at room temperature (RT). HCRSV swollen virions were digested with trypsin at RT and harvested at different time points (0, 5, 25 and 60 min, respectively), followed by separation of the digested proteins on 15% SDS-PAGE gel. (B) Limited trypsin digestion. To optimize the digestion in order to obtain the only one smaller size protein band, samples were differentially treated. Symbols “+” and “–” above each lane represent treatment with or without corresponding reagents or temperature conditions. Samples were collected immediately after each denaturation and stored in freezer, followed by separation on 12% SDS-PAGE gel, immediately after the last sample was collected. (C) Observation of trypsin digested HCRSV particles. The limited trypsin-digested swollen HCRSV virions were dialyzed by a two-step method [33], followed by concentration and observation under TEM. Each bar represents 40 nm. (D) Detection of HCRSV RNA in reassembled particles by RT-PCR with primers HC-R3, HC-F5 and HC-R5, HC-F8 and HC-R3 (Table 1) for detection of its genomic and sgRNA.

HCRSV CP was not expressed in mutant 2590 (A to G) inoculated kenaf plants (Fig. 3B). Therefore, we are unable to know whether the truncated HCRSV CP, without its N-terminal 67 amino acids including the β-annulus domain, could assemble into virions. To ascertain this, trypsin digestion of expanded (swollen) purified HCRSV virions was carried out as native capsids will open and expand after incubation in the virion swelling buffer [36] and the swollen virions are easier for proteolysis [30], [31]. Time-course trypsin treatment to swollen HCRSV virions at room temperature (Fig. 6A) showed that after 5 min of incubation, the full-length CP was partially digested to smaller size (approximate 30 kDa). After 25 min, only a faint band of the full-length CP was detected. After 1 h, no protein was observed. To avoid complete digestion, immediately after 5 min of trypsin treatment at room temperature, the reaction was carried out at 4°C overnight, resulting in a smaller size HCRSV CP band (approximate 30 kDa) (Fig. 6B, fourth lane from the left) which reacted positively with the HCRSV CP antibody (Fig. S1). N-terminal sequencing to the smaller band showed that the trypsin digestion site is located at arginine 77 of the full-length CP (Fig. S2). This indicates that the N-terminal amino acids including the β-annulus domain was removed successfully by the limited trypsin digestion.

To allow proper reassembly of the trypsin-digested HCRSV, two-step dialysis was performed [33], followed by concentration using centrifugation. The concentrated solution was used for observation of any assembled particles using TEM. Both the wt and smaller size particles were observed in the sample digested with trypsin for 5 min. However, only T = 1 particles with diameter of approximate 20 nm were observed after further trypsin treatment at 4°C overnight (Fig. 6C). HCRSV RNA was not detected in the smaller size particles (Fig. 6D).

## Discussion

In this study, we determined functions of HCRSV CP on viral RNA replication, cell-to-cell, long-distance movement and virion assembly. Unlike other carmoviruses, HCRSV encodes for additional two proteins, p27 and p25, which overlap with the CP. As a result, the N-terminal nucleotide sequence of the CP could not be deleted for its functional studies. Instead, point mutation was chosen to disrupt the CP start codon in p2590 (A to G) which theoretically would result in a CP lacking the N-terminal 67 amino acids. When the mutation site in mutant 2590 (A to G) was considered, only the A to G substitution option would not result in amino acids changes of p27, although we were aware that GTG is a weak start codon. The second ATG in-frame within the CP ORF is located at nt 2791 which is in close proximity of proline 63. The other three upstream ATGs which are in-frame with p27/25 but not in-frame with CP ORF are located at nt 2603, 2630 and 2666, respectively. Therefore, no other polypeptides are expected from this mutant. For mutant 2776 (C to G), we selected the nucleotide which does not change the amino acids in p27 and p25. Thus, the expression of p27 and p25 will not be affected.

Protoplasts transfection results showed that mutant 2590 (A to G) replicated at a similar level as compared to the wt (Fig. 2B), although the predicted truncated CP was not detected in the mutant (Fig. 2C). These results reveal that CP is not required for HCRSV replication in protoplasts. This is in agreement with the studies on TCV and *Tobacco mosaic virus*, which do not require CP for replication [37], [38]. However, *Alfalfa mosaic virus* does need CP to activate viral RNA replication [39], [40]. The CP accumulation in mutant 2776 (C to G) was lower than that in wild type (Fig. 2C). It is possible that expression of the mutant CP has been affected at the level of translation since no protein degradation was detected (Fig. 2C, 3rd lane from the left). The exact mechanism of proline63 alteration on virus pathogenicity needs to be further studied.

**Figure 7:**
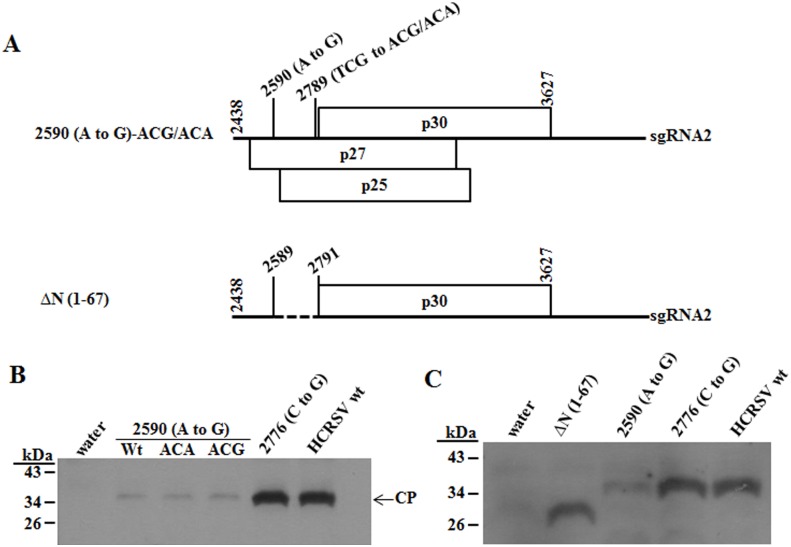
Translation of HCRSV CP and its mutants in wheat germ extract. (A) Schematic representation of HCRSV sgRNA2 mutants. p30 is an ORF encoding a putative 30 kDa protein. Mutant 2590 (A to G)-ACG/ACA represents two individual sgRNA2 mutations at ACG or ACA, respectively. The TCG in mutant 2590 (A to G) before the second in-frame ATG of CP was substituted with Kozak sequence ACG or ACA. ΔN (1–67) represents HCRSV sgRNA2 with an N-terminal deletion of CP amino acids between the first and the second ATG of the CP gene ORF and the dotted line represents the deleted nucleotides. (B & C) *In vitro* translation of HCRSV sgRNA2 and its mutants. The PCR products of the sgRNA2 of HCRSV and its mutants were used for *in vitro* transcription, followed by *in vitro* translation and the products were labeled with biotinylated lysine.

The truncated CP to be expected was not detected in mutant 2590 (A to G) transfected kenaf protoplasts and in inoculated plants (Figs. 2C and 3B). In theory, the CP would be translated from the second start codon (methionine 68) when the first start codon is mutated in the mutant 2590 (A to G). However, this may not happen in the host because of different conditions such as the absence of the Kozak sequence before the second initiation codon (ATG) which is thought to be important for translation initiation [41], [42]. To ascertain this, two additional full-length cDNA mutants were constructed with the Kozak sequence ACG or ACA added before the second ATG of CP in mutant p2590 (A to G). The CP was still not detected in the two mutants transfected protoplasts (Fig. S3). To confirm the result, PCR products covering the sgRNA2 region of the two mutants were obtained (Fig. 7A) and *in vitro* translation of their RNA transcripts showed a similar weak CP expression as that of mutant 2590 (A to G), compared to the strong CP expression in mutant 2776 (C to G) and HCRSV wt (Fig. 7B). Weak expression of CP *in vitro*, compared to the absence of CP expression *in vivo*, may be explained as: when ATG is mutated to GTG which can also serve as a start codon but with lower efficiency [43], a lower level of CP is expressed in a less competitive *in vitro* environment, whereas, in a more competitive *in vivo* environment, GTG is less efficient to compete against host factors for protein translation initiation. Based on evidence from *in vitro* and *in vivo* experiments on the presence of Kozak sequence for translation, we conclude that the lack of CP expression in mutant 2590 (A to G) is not due to the lack of Kozak sequence before the second ATG. Our second hypothesis is that the distance between the 5′ end of sgRNA2 to the second in-frame ATG is too far apart so that ribosome could not reach and bind to it for translation. To ascertain this, a mutant ΔN (1–67), covering the sgRNA2, in which the nucleotides between the first and the second ATG was deleted, was constructed (Fig. 7A). The *in vitro* translation result showed a similar level of the CP expression among ΔN (1–67), wt and mutant 2776 (C to G), while the CP expression of mutant 2590 (A to G) remained low (Fig. 7C). Therefore, we conclude that the ribosome bound to the 5′ end of HCRSV sgRNA2 of mutant 2590 (A to G) is unable to travel the distance between the first and second ATG of the CP gene to initiate translation.

Efficient cell-to-cell movement of plant virus is needed for local lesion development [34], [35]. In TCV, CP is required for cell-to-cell movement in *N. benthamiana* plants, but not in *Arabidopsis* plants [37], [44]–[46]. Small lesions have been reported to be formed in TCV inoculated *Chenopodium amaranticolor* leaves, indicating that TCV lacking CP is able to move from cell-to-cell inefficiently in *C. amaranticolor* [37]. In *Black beet scorch virus*, elimination of its sgRNA2 showed that the viral CP was not required for viral RNA accumulation or the appearance of local lesions in *C. amaranticolor* [47]. *Red clover necrotic mosaic virus* CP is also not required for its cell-to-cell movement in *N.benthamiana* and *N. clevelandii* [48]. The CPs of *Brome mosaic virus* (BMV) and *Cucumber mosaic virus* (CMV), functioning together with their MPs, are reported to be essential for their virus cell-to-cell movement, as virion and non-virion forms, respectively [49], [50]. BMV or CMV with Deletion or mutation of their MP C-terminal amino acids move from cell to cell independently of their CPs [51], [52]. In *Wheat streak mosaic virus*, mutation in the conserved amino acids in the core domain of its CP abolished virion assembly and cell-to-cell movement [35]. In HCRSV mutant 2590 (A to G) inoculated cotyledons, the viral RNA level did not increase over time (Fig. 3A), and both CP (Fig. 3B) and local lesions (Fig. 3C) were not detected. These results clearly demonstrate that HCRSV CP is essential for its virus cell-to-cell movement. Perhaps HCRSV moves intercellularly with help of its CP and MP as nucleoprotein complex, similar to CMV. Exactly how HCRSV CP is involved in its cell-to-cell movement remains to be studied.

Functional CP or formation of stable virion have been shown to be essential for long-distance movement in many plant RNA viruses such as alfamoviruses [53], cucumoviruses [54], dianthoviruses [55], potyviruses [56], sobemoviruses [57] and tobamoviruses [58], [59], except for a few cases [60]–[62]. In TCV, its CP plays a host-dependent role in the virus long-distance movement [37], [45], [63], [64]. The proper virion assembly in *Beet black scorch virus* (BBSV) is needed for the virus systemic movement [65]. In *Olive latent virus 1*, the CP C-terminal mutants could form intact virions but its systemic movement was not detected, indicating that virion formation is necessary but not sufficient for long-distance movement [66]. In HCRSV, virions were not observed in mutant 2776 (C to G) inoculated cotyledons when proline 63 was replaced by alanine (Fig. 4A). Subsequently, systemic infection in plants was not observed (Fig. 5). This indicates that HCRSV requires virions to establish systemic infection in kenaf plants. In the partially purified virus particle samples isolated from mutant 2776 (C to G) inoculated cotyledons, significant amount of CP was detected (Fig. 4B), although virions were not observed. Virions were readily observed under TEM in wt samples diluted five folds (data not shown), although the amount of CP was less than that from mutant 2776 (C to G) samples without dilution (Fig. 4B). There is no evidence for the presence of virions, despite thorough search for virions of mutant 2776 (C to G) sample under TEM. It is possible that CP is pelleted together with HCRSV RNA as a RNA-CP complex after ultra-high speed centrifugation. Virions were only observed in wt virus samples but not in mutant 2776 (C to G) samples regardless of using relative high or low pH (Fig. 4A) buffer in sucrose cushion solution and resuspension buffer for virion purification. This result shows that absence of the virions in mutant 2776 (C to G) is not due to the relative high pH used for virion purification which may render in less stable virions. In BBSV, the CP was detected but not the virions in the purified virion samples isolated from a CP N-ARM mutant BM5 inoculated plants and the authors postulated that it was due to lower level of virion assembly [65]. In this study, despite exhaustive search for virions, there is still no evidence to show that lack of virions in proline 63 mutant inoculated plants is due to low level of virion assembly.

In SeMV, replacement of the equivalent and conserved proline 53 with alanine can still form T = 3 VLPs *in vitro* [20]. The bending and the β-annulus structure seem not to be affected [21]. Similar replacement of the proline 85 with glycine in CNV results in T = 3 and T = 1 particles in *vivo*, although the particles yield is only 0.4% of the wt [22]. In bacteriophage MS2, replacement of the conserved proline 78 with asparagine allows the expressed CP in *E. coli* to form T = 3 VLP [23]. However, the same mutation in the full-length cDNA clone of MS2 abolished the production of infectious particles [24]. In this study, no virions were observed in the proline 63 mutant [2776 (C to G)], inoculated cotyledons (Fig. 4A), although it is possible that the mutant virions were present at an extremely low amount. Similar to the proline 78 in MS2 and proline 85 in CNV, the proline 63 of HCRSV plays an important role in virion assembly *in vivo*.

A T = 1 VLP has been detected in trypsin digested BMV which removed the N-terminal 63 amino acids of the CP [67]. HCRSV, unlike other carmoviruses, has a unique ORF encoding a p27 protein and a p25 protein which overlap with the CP ORF [68]. Absence of these two proteins will abolish viral movement and CP expression in kenaf plants. Therefore, we could not delete N-terminal region of HCRSV CP to study its effects on HCRSV assembly in plants. To overcome this, limited trypsin digestion strategy was used to obtain CP with its N-terminal region deleted. Small T = 1 empty VLPs with diameter approximate 20 nm [69] were observed under TEM (Fig. 6C). This result reveals that the N-terminal 77 amino acids in HCRSV is essential for T = 3 virion assembly *in vitro*. In the 77 amino acids, we did not find N-terminal ARM which exists in other related viruses and is involved in T = 3 virion assembly [65], [70]. Based on the available information on HCRSV structure, the R-domain is from amino acid 1 to 99. Perhaps the lack of viral RNA in the HCRSV T = 1 VLPs is due to the loss of RNA binding of the CP lacking the N-terminal 77 amino acids.

In conclusion, our study showed that HCRSV CP is not required for HCRSV RNA replication but essential for cell-to-cell movement. Assembled virions are crucial for long-distance movement. The proline 63 is essential for virion assembly in *vivo* and the CP N-terminal 1–77 amino acids are important for T = 3 particles formation *in vitro*.

## Supporting Information

Figure S1 HCRSV virions digested with trypsin to remove N-terminal 1–77 amino acids reacted with antibody against HCRSV virions. HCRSV virions without trypsin digestion were used as a control.
(TIF)

Figure S2 N-terminal sequencing of trypsin digested HCRSV CP. HCRSV virions were limited digested by trypsin and concentrated, followed by transferring onto PVDF membrane. A protein band (approximate 30 kDa) shown on the membrane after commassie blue staining was cut out for N-terminal sequencing and the results of first three sequencing cycles were shown.
(TIF)

Figure S3 HCRSV CP accumulation in transfected kenaf protoplasts. *In vitro* transcripts (10 µg each) were transfected into 9×10^5^ protoplasts and harvested at 72 hpt for total protein extraction. The TCG in mutant 2590 (A to G) before the second in-frame ATG of CP was substituted with Kozak sequence ACG or ACA. wt, ACG and ACA represent HCRSV full-length cDNA clone mutants 2590 (A to G), 2590 (A to G)-ACG and 2590 (A to G)-ACA, respectively.
(TIF)
