# Supplementary figures and images for: Hibiscus Chlorotic Ringspot Virus Coat Protein Is Essential for Cell-to-Cell and Long-Distance Movement but Not for Viral RNA Replication
Source: PLoS One. 2014 Nov 17;9(11):e113347. doi: 10.1371/journal.pone.0113347 (PMC4234673; doi:10.1371/journal.pone.0113347)

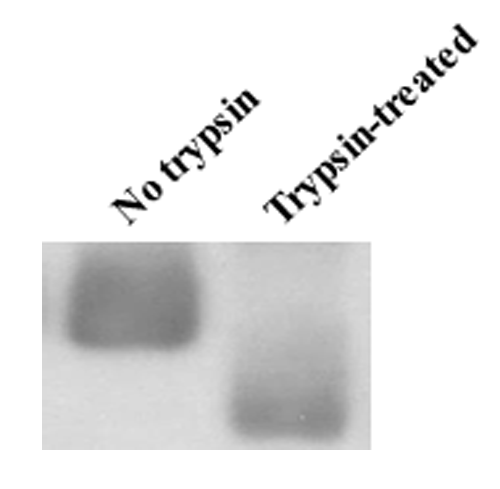

Supplement: Figure S1 — HCRSV virions digested with trypsin to remove N-terminal 1–77 amino acids reacted with antibody against HCRSV virions. HCRSV virions without trypsin digestion were used as a control. (TIF) [file pone.0113347.s001.tif]

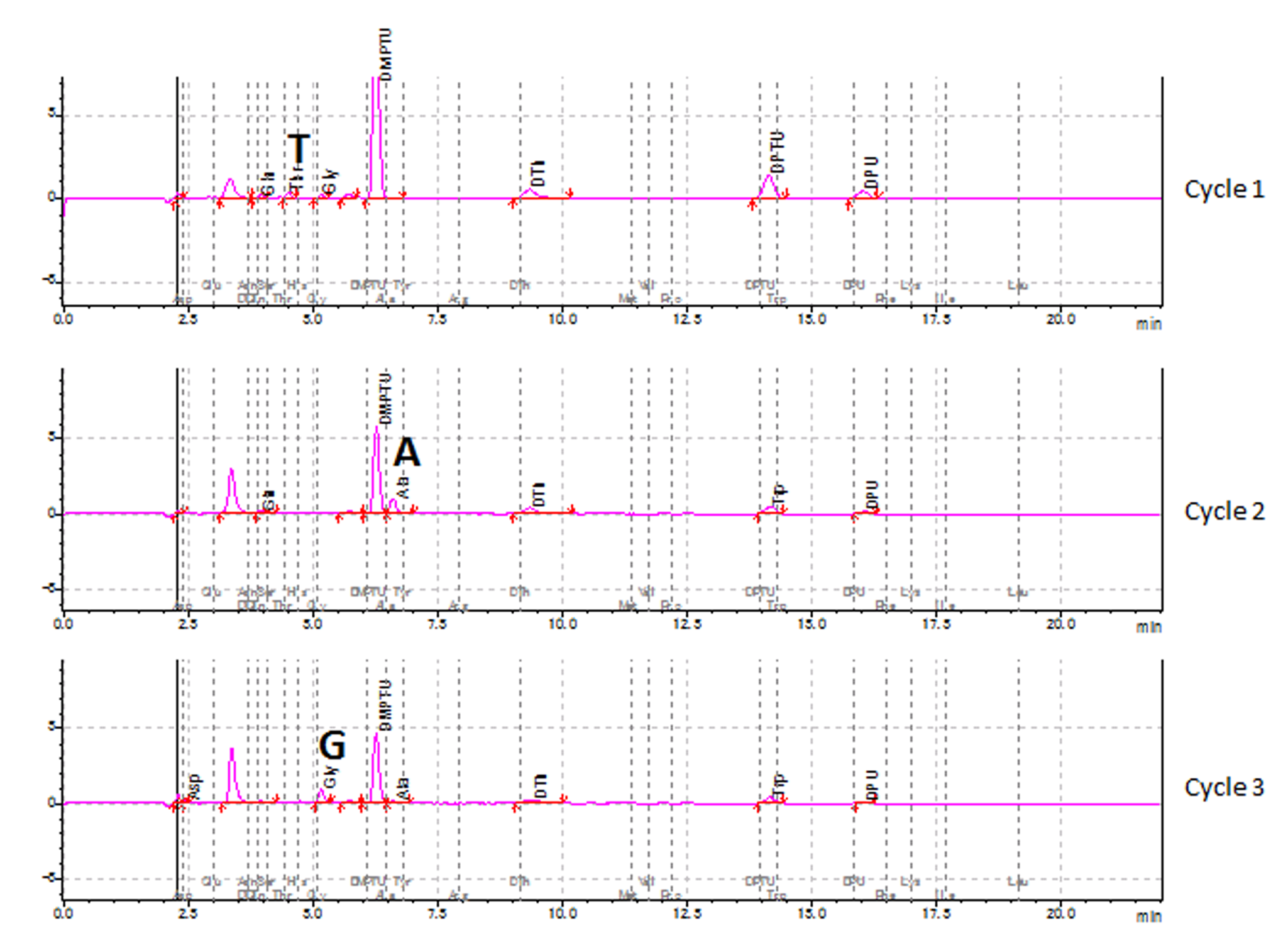

Supplement: Figure S2 — N-terminal sequencing of trypsin digested HCRSV CP. HCRSV virions were limited digested by trypsin and concentrated, followed by transferring onto PVDF membrane. A protein band (approximate 30 kDa) shown on the membrane after commassie blue staining was cut out for N-terminal sequencing and the results of first three sequencing cycles were shown. (TIF) [file pone.0113347.s002.tif]

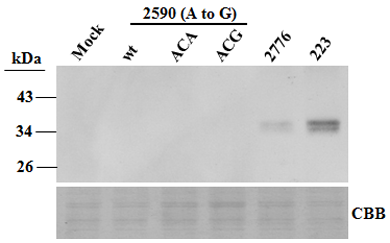

Supplement: Figure S3 — HCRSV CP accumulation in transfected kenaf protoplasts. In vitro transcripts (10 µg each) were transfected into 9×10^5^ protoplasts and harvested at 72 hpt for total protein extraction. The TCG in mutant 2590 (A to G) before the second in-frame ATG of CP was substituted with Kozak sequence ACG or ACA. wt, ACG and ACA represent HCRSV full-length cDNA clone mutants 2590 (A to G), 2590 (A to G)-ACG and 2590 (A to G)-ACA, respectively. (TIF) [file pone.0113347.s003.tif]
